# Integrated prediction of one-dimensional structural features and their relationships with conformational flexibility in helical membrane proteins

**DOI:** 10.1186/1471-2105-11-533

**Published:** 2010-10-27

**Authors:** Shandar Ahmad, Yumlembam Hemajit Singh, Yogesh Paudel, Takaharu Mori, Yuji Sugita, Kenji Mizuguchi

**Affiliations:** 1 National Institute of Biomedical Innovation, 7-6-8, Saito-asagi, Ibaraki, Osaka 567 0085, Japan; 2 Institute of Bioinformatics Research and Development, Japan Science and Technology Agency (JST-BIRD), Japan; 3 Advanced Science Institute, RIKEN, 2-1, Hirosawa, Wako, Saitama 351-0198, Japan

## Abstract

**Background:**

Many structural properties such as solvent accessibility, dihedral angles and helix-helix contacts can be assigned to each residue in a membrane protein. Independent studies exist on the analysis and sequence-based prediction of some of these so-called one-dimensional features. However, there is little explanation of why certain residues are predicted in a wrong structural class or with large errors in the absolute values of these features. On the other hand, membrane proteins undergo conformational changes to allow transport as well as ligand binding. These conformational changes often occur via residues that are inherently flexible and hence, predicting fluctuations in residue positions is of great significance.

**Results:**

We performed a statistical analysis of common patterns among selected one-dimensional equilibrium structural features (ESFs) and developed a method for simultaneously predicting all of these features using an integrated system. Our results show that the prediction performance can be improved if multiple structural features are trained in an integrated model, compared to the current practice of developing individual models. In particular, the performance of the solvent accessibility and bend-angle prediction improved in this way. The well-performing bend-angle prediction can be used to predict helical positions with severe kinks at a modest success rate. Further, we showed that single-chain conformational dynamics, measured by B-factors derived from normal mode analysis, could be predicted from observed and predicted ESFs with good accuracy. A web server was developed (http://tardis.nibio.go.jp/netasa/htmone/) for predicting the one-dimensional ESFs from sequence information and analyzing the differences between the predicted and observed values of the ESFs.

**Conclusions:**

The prediction performance of the integrated model is significantly better than that of the models performing the task separately for each feature for the solvent accessibility and bend-angle predictions. The predictability of the features also plays a role in determining flexible positions. Although the dynamics studied here concerns local atomic fluctuations, a similar analysis in terms of global structural features will be helpful in predicting large-scale conformational changes, for which work is in progress.

## Background

Membrane proteins are essential to intercellular transport and communication and they constitute among the most important drug targets. Since the majority of membrane proteins adopt helical conformations, this type of membrane proteins has been studied extensively and in this work, the term *membrane proteins *refers to helical membrane proteins unless otherwise stated. Due to the difficulties in protein production and crystallization, complete structures of very few of these proteins have been determined[1]. To overcome the limitations in the number of available structures, comparative modeling is useful but may not always be feasible because of unavailable templates[2].

As an alternative and complementary approach, it has been found useful to try to estimate lower resolution structural features, rather than the whole structure, from amino acid sequence information alone. This has led to methods for predicting transmembrane (TM) regions[3,4], solvent accessibility[5-7], and helix-helix contacts (HHCs)[8-10]. Most prediction studies focus on a single (often one-dimensional) residue-wise property of proteins and develop an analysis and prediction method around it. To construct prediction models, a variety of techniques have been used, such as artificial neural networks, support vector machines and hidden Markov Models. The basic underlying principle in these techniques is that a target structural property such as the solvent accessibility of a residue depends on the local sequence features, such as the identity of the residues in given positions relative to the residue under consideration. The purpose of a computational model is to determine a general relationship between these sequence features and the target property. Sequence features are often taken from the evolutionary profile of residues in the vicinity of each residue. Sometimes global features of protein sequence, such as its amino-acid composition and sequence length, are also considered, which can improve prediction performance[11,12].

These methods have been successful in predicting solvent accessibility, TM regions and helical contacts with fair degrees of accuracy. For example, the prediction of solvent accessibility has been performed with a mean absolute error of ~19%[5], HHC with a ~78% accuracy[9] and TM regions with an 89% accuracy[4]. Although estimates of the performance of a prediction method depend on the data sets, cross-validation procedures and performance score used, the numbers quoted above give us a general idea of the status of prediction efforts for the structural features attempted so far.

It has been reported in the case of globular proteins that adding predicted values of one structural feature (secondary structure) improves the performance of predicting another feature (solvent accessibility)[13]. This observation motivated us to consider developing an integrated system for predicting several structural features of membrane proteins simultaneously. Another reason for predicting many new structural features is that we wish to understand how these (predictable) structural features are related to each other and to the dynamics of membrane protein structures.

While most of the existing prediction methods deal with static structural properties, membrane proteins have dynamic structures, required for their channeling and gating type functions[14]. Deriving the knowledge of dynamic structures, either from a single known structure or from a sequence, is challenging and not always possible. Molecular dynamics (MD) simulations have been performed for some membrane proteins [15-17]. However, all-atom simulations are time-consuming and even coarse-grained simulations can provide limited information about large-scale conformational changes [18]. On the other hand, very few ab initio-type structure prediction methods have been developed for membrane proteins and knowledge-based approaches largely depend on the availability of templates, e.g., multiple crystal structures solved in different conformations.

In this paper, we wish to discuss both static and dynamic structural features of membrane proteins within the framework of the prediction of residue-wise properties from amino acid sequence information alone. As we mentioned above, many prediction methods have been developed but so far, there has been no attempt to integrate predictions of multiple one-dimensional features, which are expected to share some common patterns and even influence one another. The first component of this study addresses this issue and develops an integrated system to predict multiple one-dimensional structural properties from amino acid sequence and evolutionary information using a well-established technique for sequence-based predictions. Mutual dependencies of errors in these features are also explored. Due to the relatively static nature of these one-dimensional features within a structure, we term them one-dimensional equilibrium structural features (ESFs), even though they may not represent equilibrium in the strict sense.

In the second component of this study, we try to determine whether the observed or predicted ESFs may be used to estimate the dynamic nature of structures. We derive a data set of atomic fluctuations estimated from a normal modes analysis (NMA) and represented by a theoretical B-factor and try to predict these B-factor values from sequence and structural features directly. We show that the B-factor values derived from the NMA can be predicted from the observed and predicted values of ESFs. A small data set of MD-trajectories of proteins suggests that these predictabilities extend to the local dynamics at a larger time scale.

## Methods

### Data set

A non-redundant data set of helical membrane proteins (selected at a chain level) was developed as follows. First, protein chains from PDBTM [19] were downloaded as on April 4, 2010. From the initial list of 286 protein chains in pdbtm_alpha_nr.seq, entries containing modified residues or with a resolution poorer than 4Å were removed. Sequence similarity was reduced by running the BLASTCLUST program and selecting chains such that no two chains shared more than 25% identity[20]. This resulted in 76 polytopic proteins (Table S2; Additional File 1). This dataset is referred to as TM76 in this work. TM regions were taken from PDBTM, and the distance from the bilayer plane was computed from the z-coordinate values of the corresponding entries in OPM [21].

### One-dimensional equilibrium structural features (ESFs)

Unlike earlier studies predicting the residue-wise solvent accessibility and helix-contacting status separately, we study, in this paper, eight one-dimensional structural features in an integrated manner. Since all these structural features are calculated from crystal structures, which represent a good estimate of the equilibrium state of at least one of the available conformations, we call these features equilibrium structural features (ESF). In the strict sense these features may not represent equilibrium, but none of the results presented in this work are likely to be affected by this approximation. The first three features represent the solvent accessible surface area (ASA), followed by the HHC status and four different conformational angles. These eight features are calculated/defined as follows:

#### Solvent accessibility (three mutually redundant features)

ASA values were computed using the program NACCESS[22], with the atomic coordinates of isolated target chains. The default water probe was replaced by a hypothetical lipid probe with a sphere radius of 1.9Å. This probe size has been widely used [23] to estimate lipid accessibility of TM residues, as against water-accessibility for which the probe radius is typically set to 1.4Å. Relative ASA values, normalized by the ASA of an extended state, were used for all our analysis. NACCESS calculates atomic ASA values and groups them together by the atom type in five different ways. Only three of these values are independent and in this study, we defined the following three ASA features for each amino acid residue: 1) residue total (tASA), 2) side chain total (scASA) and 3) non-polar total (npASA). This set of structural properties has been widely studied for water-soluble and membrane proteins and more recently for DNA and RNA structures [24,25].

#### Helix-helix contact (one feature)

We assigned each TM residue a binary value of 1 or 0, depending on whether it is in contact with another TM helix. A contact between an inter-helical pair of residues was defined if any atom from one residue was within a cut-off distance from any atom from the other residue. The distance threshold for this study was chosen to be 3.5Å after analyzing the distribution of the closest inter-helical distances (see Results). Only intra-chain contacts were included, as the atomic coordinates of isolated chains were used throughout.

#### Backbone conformational angles (four features)

Four conformational angles, kappa (κ), alpha (α), phi (φ) and psi (ψ), were considered, as defined and calculated by the dictionary of secondary structure of proteins (DSSP)[26]. In brief, kappa (κ) or the bend angle refers to the angle defined by the three *C_α _*atoms of residues *i-2*, *i *(the target) and *i+2*. This angle provides an estimate of the local bend in the peptide chain of the protein. Alpha refers to the helical torsion angle defined by the four *C_α _*atoms of residues *i-1, i, i+1 *and *i+2 *and approximates the chirality, whereas phi and psi are well known dihedral angles.

#### Additional information (kink angles)

The bend angle defined above is a property of peptide chain conformation and has the advantage that each position can be assigned a finite and unambiguous value. In some studies, the bend angle is also referred to as the angle between the helical axes over the residues in preceding and following parts of a helix. There is no unique definition of helical axes when a bend is present, but we used the definition of Bansal and Kumar, and performed this analysis using their software HELANAL [27,28]. To distinguish this from the DSSP-derived bend angle defined above, we call this angle the kink angle throughout this paper. This angle does not form a separate structural feature in the prediction target vectors because it requires a definition of helical axes and is undefined near helical boundaries, but the kink angle was used in discussing the prediction performance for the DSSP-derived bend angle. Helical axes for the purpose of this work are computed by considering four residue helices on either side of the residue position, for which the kink angle is calculated, making it a 9-residue running window for all helical positions, except near the membrane boundaries.

### Dynamic structural features (DSFs)

In contrast to the residue-wise ESFs considered above, dynamic structural features (DSFs) are defined by real-time displacements from the equilibrium state and cannot be calculated directly from the atomic coordinate data of crystal structures. As DSFs, we considered the root mean square fluctuations (RMSF) of the *C_α _*atoms, estimated by two techniques, NMA and MD simulations.

#### NMA

In NMA, the energy surface is assumed to be harmonic and atomic displacements are expressed as a superposition of linearly independent collective motions called normal modes. We carried out the normal mode analysis of all the proteins in our data set using the El Nemo server[29,30]. This web server uses a simplified force field with single parameters to estimate interaction energies between residues/atoms, which has been reported to calculate the mean atomic displacements with reasonable accuracy[31]. For our analysis, we used the calculated B-factors directly obtained from this server.

#### NMA-derived fluctuations and position along the helical axis

Although the normal modes and displacements were calculated for an entire protein chain, all the analysis of relationships between ESFs and B-factors was performed for the membrane-spanning regions only. Residues close to the membrane boundaries are expected to be more mobile than those inside and thus a baseline performance needed to be estimated. To do so, all the residues were assigned a helical z-axis position taken from OPM and a correlation between the values of the z-coordinate and the NMA-derived B-factors (BNMA) was computed. This correlation serves as the baseline indicating the extent to which fluctuations can be determined from the knowledge of TM topology alone.

#### Molecular Dynamics trajectory data

Unlike NMA, MD simulations make no assumption about the energy surface and therefore, are expected to provide more accurate estimates of DSFs. In addition, they represent a larger conformational space compared to normal modes or static crystal structures. However, all-atom MD simulations are time consuming and it is not possible to obtain the trajectory data for all the proteins in our data set. We, therefore, used a smaller data set of proteins, for which MD data became available during the course of this work. Due to the small number of examples available, this part of the work serves as an example application of the current methodology rather than providing a statistical rule.

The proteins used in this study are the protein-conducting channel SecYE from *Thermus thermophilus *(ttSecYE; PDB code: 2ZJS[32] and SecYEβ from *Methanococcus jannaschii *(mjSecYEβ; PDB code: 1RH5)[33,34]; ttSecYE and mjSecYEβ consist of two chains (SecY and SecE) and three chains (SecY, SecE, and Secβ), respectively. MD trajectory data were obtained from the all-atom model simulations of these proteins in the fully hydrated POPC bilayers using the isothermal-isobaric ensemble (NPT) and constant area isothermal-isobaric ensemble (NPAT)[32,34]. The total simulation length was 100 ns for each simulation run, and the coordinates saved every 10 ps were used for the calculation of the RMSF.

We used observed and predicted ESFs, as well as their combination, to estimate the MD-derived RMSF values, i.e., the fluctuations that take place over a longer time scale, much in the same way as we did for BNMA predictions.

### Prediction Method

#### Prediction of one-dimensional ESFs: Individual versus integrated models

The prediction of multiple features can be achieved either by training models for each feature separately or in a single integrated system. The integrated predictions allow for information flow from the prediction of one feature to another and hence may be beneficial for performance reasons. In addition, the number of training/validation cycles in an integrated system is substantially fewer than that of models for individual features. However, at the outset, it is not clear if the integration results in an improved performance or various components of the target features in the integrated model interfere with each other and cause a performance loss. To find a definite answer to this question, we trained separate models for each of the eight features considered and compared the performance between the individually trained models and the integrated model.

In general, the prediction models were similar to that of our recent work on predicting multidimensional properties corresponding to the bound dinucleotide identities in DNA-binding proteins[11]. To summarize, all predictions for ESFs were performed using a multilayer feed forward neural network, whose inputs are the position-specific scoring matrix (PSSM) rows of the target residue and its eight sequence neighbors, calculated by PSI-BLAST[20] with default parameters for three iterations. The overall amino acid composition of the protein was also included as an additional feature of each residue. The output or target was a feature vector formed by the eight structural properties, defined above and scaled to values between 0 and 1. The number of hidden units in a single hidden layer was consistently kept at twice the number of features to be predicted (16 for the integrated model and 2 for the individual models). Prima-facie it may appear that the integrated model has more (8) hidden units and perhaps a comparison with less complex, individually trained model (with only 2 hidden units) is unfair. However, it should be noted that there are eight independent neural networks in the individually trained models, whereas there is only one model in the integrated version. The total number of trainable parameters (network weights) in the two cases is, therefore, almost identical, the difference being only about 3%, which arises from the additional connections as a result of the integration, and not from the higher complexity of the integrated model. Thus, it is fair and justified to compare the two prediction methods by keeping the number of hidden units per feature equal. The neural network simulation and training were performed using the SNNS software[35]. To avoid over-fitting, the leave-one-out (LOO) method of training was used such that all but one protein were used to train the network for a fixed number of epochs while one protein was left out of training. After the training was completed, the performance was evaluated on the left-out protein. All possible combinations of leaving one-protein out were trained independently, so as to obtain the prediction performance for all the proteins in the left-out state. All the prediction performances reported in this paper correspond to proteins in this left-out state and therefore, represent the true generalization value of the neural networks without over-fitting.

ESF predictions for the proteins with MD trajectory data were performed by using one of the 76 neural networks trained above, where the left-out protein showed the highest sequence similarity with the query protein. This procedure ensures that the prediction of ESFs was made by a model trained on truly dissimilar (< 25%) sequences, eliminating a bias causing exaggerated prediction performance.

#### Prediction of TM regions

In this work, we do not intend to develop a method for predicting TM regions, as a number of methods for doing so are available[4,12,36-38]. However, since we provide a web server for sequence-based predictions of structural features, it may be helpful to have at least a rough estimate of TM regions. Thus, we developed a neural network for predicting TM regions using the same cross-validation (LOO) procedure. The training and neural network implementation was similar to that used for our earlier method to predict TM regions in beta-barrel proteins[39]. Using similar neural networks with no post-training refinements, we obtained a high performance of neural network (AUC~0.88). Thus, even though the prediction of TM regions on the web server is provided only for guidance purposes, the regions labeled TM may be expected to be ~88% accurate.

#### Prediction of dynamic structural features (DSFs)

To evaluate the relationships between ESFs and DSFs, we calculated the coefficients of correlation between each of the eight ESFs and the BNMA for the individual proteins. To determine the role of cooperativity between various features, neural networks were trained, using various combinations of feature sets as inputs, to predict the BNMA values of all the residues in the membrane-spanning regions.

Further, the observed and predicted ESFs were used in various combinations to predict the MD-derived RMSF values. Since the amount of data is small for an LOO validation here, all the samples were pooled to train the neural network. To avoid overfitting on the training samples, the background performance of a random data was computed in the following way. The RMSF values were randomly redistributed amongst all the residues and an attempt was made to predict these randomized values from the same feature set, as in the real data. These randomized correlations serve as a background, subtracting which from the trained correlation in the real data ensures that the performance in each case is not the result of simply an increased number of features and allows us to compare performance levels between models using different feature sets.

Also, due to the limited amount of MD data, we analyzed the entire protein including the residues outside the membrane (see Results). Since the membrane spanning regions are likely to be less flexible, a method trying to predict the RMSF over the entire protein may end up separating only the TM and non-TM regions. To estimate this effect, we labeled all the residues with a binary number (0 for membrane-spanning and 1 for non-membrane-spanning) and computed the correlation between this class label and the MD-derived RMSF. This gives background values, against which the ESF-based predictions can be compared.

### Performance evaluation

Both the analogue (solvent accessibility and conformational angles) and binary (HHC) features were predicted by defining a multidimensional output function represented by the optimized neural network. Following the standard convention, our neural networks were trained to optimize a cumulative mean squared error (MSE) defined by

(1) $$MSE=\frac{1}{N_{res}*N_{f}}\sum_{i,j}{\left(O_{ij}-P_{ij}\right)}^{2}$$

where N_res _is the total number of amino acid residues in the training data set, N_f _the total number of features (eight) and O_ij _and P_ij _are the observed and predicted values of *j^th ^*feature of *i^th ^*residue, respectively. The summation was over all the residues in all the proteins in the training data set.

MSE was used only for optimizing the neural networks. To compare performances, we used the following more widely reported measures.

#### Absolute error and Mean absolute error

Absolute error in *j^th ^*feature of a single residue is defined as

(2) $$AE(j)=|O_{j}-P_{j}|$$

Mean absolute error (MAE), which is the mean of absolute errors over all the residues, was calculated for each protein, such that MAE for the *j^th ^*feature in k^th ^protein was given by:

(3) $$MAE(j,k)=\frac{1}{N_{k}}\sum_{i}\left|O_{kij}-P_{kij}\right|$$

where N_k _is the total number of amino acid residues in k^th ^protein and *O_kij _*and *P_kij _*are, respectively, the observed and predicted values of *j^th ^*feature of *i^th ^*residue in k^th ^protein.

When referring to the MAE of the entire data, we used the average of all the protein-wise MAE values, which may be slightly different from the MAE computed by pooling together all the residues from all the proteins and computing their average error.

#### Sensitivity, specificity and AUC

For the binary valued feature of HHC, the expected (or target) values of the feature are binary (contact representing the positive class P, or no contact representing the negative class N) but the predicted values are analogue between 0 and 1. These analogue values can be translated into binary class labels by using a continuous cutoff, leading to different values of sensitivity and specificity, defined for every selected cutoff *m *and for every protein *k *as:

(4) $$Sensitivity(k,m)=\frac{TP(k,m)}{TP(k,m)+FN(k,m)}$$

(5) $$Specificity(k,m)=\frac{TN(k,m)}{TN(k,m)+FP(k,m)}$$

where k is the protein identifier, TP represents the number of true positive cases (helical contacting residues, which are predicted to be contacting), TN is true negative (non-contacting residues predicted correctly), FP is false positive (the number of non-contacting residues predicted wrongly as contacting) and FN is false negative (the number of contacting residues wrongly predicted as non-contacting). By changing the cutoff (m) systematically, a set of sensitivity versus specificity values may be obtained.

An ROC curve was plotted between sensitivity and (1-specificity) and the area under this curve (AUC) was used as a measure of prediction performance in the selected protein, over the entire range of cutoffs. The overall performance was measured by averaging the AUC values from the cross-validated prediction for all the proteins.

#### Coefficient of correlation

The prediction performance for the DSFs was measured by calculating the correlation coefficient (C) between all (n) predicted (X) and observed (Y) values of the corresponding feature as follows:

(6) $$C=\frac{n\sum X_{i}Y_{i}-\sum X_{i}\sum Y_{i}}{\sqrt{n\sum X_{i}^{2}-{\left(\sum X_{i}\right)}^{2}}\sqrt{n\sum Y_{i}^{2}-{\left(\sum Y_{i}\right)}^{2}}}$$

#### Performance comparison (t-test)

Performance of two models (for example, the integrated versus an individual model) was compared by calculating the performance measure (e.g., MAE or AUC) for each protein providing a pair of sets of performance scores corresponding to the two models. Distributions of the performance scores from the two models were compared by using a t-test implemented in the R-programming language. For all the analyses paired two-tailed t-test was used returning a p-value for the comparison, which is a measure of the evidence against the null hypothesis that the two means are equal.

## Results

### General data features

Overall, we considered 76 protein chains for this analysis. Figure S1 (Additional File 1) shows the frequency histograms of the eight ESFs considered. We have made several observations from these data. First, like water-soluble proteins, a large number of residues in these proteins are buried, a trend observed for the total (tASA) as well as the side chain (scASA) and non-polar (npASA) groups. Mean values for tASA, scASA and npASA are found to be 24%, 30% and 29%, respectively. Second, the frequency histogram of the closest inter-helical distance (the last plot of Figure S1; Additional File 1) shows that most frequently, HHCs take place at 3-4Å. For this reason, a distance threshold to label helix-helix contacts was selected to be 3.5Å. Third, the distributions of the bend angle (kappa), the chirality angle (alpha), and Phi and Psi are relatively more normal. Mean values as well as modal frequencies for these conformational angles are observed to be close to 111°, 51°, -66° and -40°, respectively.

Table S1 (Additional File 1) shows the mutual coefficients of correlation between all the ESFs considered. As expected, the three types of ASA are strongly correlated with each other (with the correlation coefficients > 0.96 in all cases) and they also show a strong negative correlation with the intra-chain HHC property. However, the highest correlation between HHC and ASA reaches only up to 0.36, with the side chain group showing the best correlation, followed by non-polar and total. This is because contacts are defined at an atomic scale, whereas all three types of ASA measure properties associated with a set of atoms. Thus, there remains significantly independent information between the two sets of features.

The four conformational angle features are poorly correlated with ASA or HHC and their correlations between each other are also weak. For example, the best correlation is 0.260 between the chirality angle (alpha) and the bend angle (kappa), both of which represent deviations from an ideal helix. The moderate level of correlation indicates that these structural features are significantly independent of each other.

### Prediction of one-dimensional equilibrium structural features (ESFs)

#### Comparison between the integrated and individually trained models

Table 1 shows a comparison of the prediction performance between the models trained individually and in an integrated manner. We observe that all the solvent accessibility features show a statistically significant performance improvement in the integrated model. A similar improvement is also shown in the bend angle Kappa (p-values ~0). However, HHC and the other conformational angles do not show any statistically significant difference between the two models (p-value ≥ 0.26 in all cases). Thus, we conclude that the prediction using an integrated model allows information flow between the features and improves the performance overall. However, some features, notably HHC, are not benefitted by the integration. Presumably, HHC depends on the global features such as the number and nature of other helices present in the protein, whereas ASA and the bend angle may be at least partially affected by each other. The other conformational angles are poorly predicted and do not seem to be strictly sequence- or PSSM-dependent, which may explain the lack of improvement in their prediction performance.

**Table 1:** Comparison of the performance between the models trained individually and in an integrated manner.

Feature	AMAE (Individual)	(SD)	AMAE (Integrated)	(SD)	p-value
tASA:	18.73	0.92	17.43	0.75	< 2.2e-16

scASA:	24.32	0.79	22.32	0.96	< 2.2e-16

npASA	22.44	1.2	22.00	1.01	0.02

Phi:	12.16	0.77	12.13	0.74	0.82

Psi:	12.55	0.61	12.53	0.6	0.82

Kappa:	8.51	0.57	8.20	0.51	< 2.2e-16

Alpha:	10.21	0.64	10.23	0.63	0.84

HHC(AUC):	67.04	1.58	67.29	1.19	0.26

AMAE represents the average of the protein-wise mean absolute error (or AUC, the area under the ROC curve, in the case of HHC) and the standard deviation (SD) indicates the protein-wise variation of the AMAE values. The p-values are obtained by Welsh's t-test implemented in the R-programming language. The Integrated model outperformed the individually trained ones for the ASA and bend angle (kappa) predictions, but showed no statistically significant difference for the other conformational angles.

#### Comparison with publicly available web servers

To make a comparison with other publicly available methods of prediction, we identified three web servers, two of which (MPRAP and ASAP) successfully returned ASA-predictions [5,40], whereas another (RHYTHM) returned HHC predictions[41]. Some of the proteins, for which one or more of these servers failed to return any prediction results, were eliminated from the comparison. The results were compared in terms of MAE for ASA (tASA in our case) (Figure S2; Additional File 1). For the HHC prediction, a binary value was returned by RHYTHM, which was converted to specificity and sensitivity scores and the point representing this performance is shown on the ROC plot of our method (Figure S3; Additional File 1).

The overall average MAE-values for MPRAP and ASAP are 15% and 21%, respectively. On the other hand, our LOO cross-validated integrated model has an MAE of 17%, whereas a self-consistently trained integrated model (used in the HTM One web server described below) has the lowest MAE of 14.2%. Since web servers are likely to be based on a model that is trained with all the proteins available at the time of the study and hence, the servers' performance will be exaggerated for the proteins used in the training. Our own web server performs about 3 percentage point better than the cross-validated performance on the considered proteins. ASAP was one of the first servers of this kind and is likely to have used fewer proteins. MPRAP has been developed recently and is based on more data, explaining why the latter performs better on the selected proteins. The cross-validated performance of our method is slightly lower than the result from the MPRAP server, but is more likely to represent a performance on totally new proteins.

In summary, we conclude that the performance of our integrated approach, which was shown to outperform the individually trained models under identical conditions, is also likely to be better than currently available web servers.

#### Performance and error analysis of integrated prediction model

**Figure 1:**
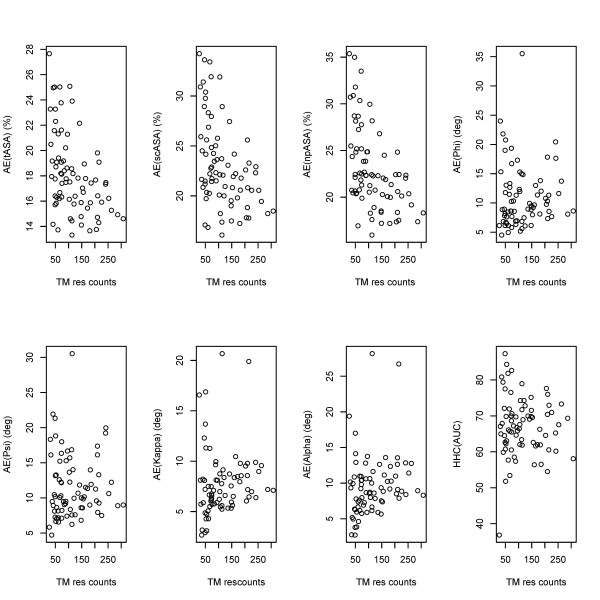
**Performance of the integrated prediction model for various equilibrium structural features as a function of the membrane spanning length of the protein (AE: Mean Absolute error in percentage points; TM res counts: the number of residues of a protein in the membrane spanning region)**. Each point in the plot represents one protein.

Tables 2 and S2 (in Additional File 1) summarize the average and protein-wise cross-validation performance results, respectively, when the integrated model was trained. Generally, the performance of the ASA prediction appears to depend on the number of residues in the TM regions (Figure 1; with additional information in Table S2 of Additional File 1). This trend is not observed for the conformational angles, which are generally less well predicted. The better performance of the ASA prediction for proteins with larger number of TM residues can be explained as follows. The prediction performance measured by cross-validation would represent the degree to which a given protein approximates the average behavior of these features in the training data (i.e., all the proteins excluding itself). Thus, a high prediction error would indicate that the protein has unique structural features. Small proteins with a few helices have more exposed residues, an unusual feature that is difficult to predict. Larger proteins have more buried residues and thus lead to better performances for the ASA predictions.

**Table 2:** Summary of the correlation and mean absolute error (MAE) between the predicted and observed values of the residue-wise structural features.

	tASA (%)	scASA (%)	npASA (%)	Phi (deg)	Psi (deg)	Kappa (deg)	Alpha (deg)	HHC (%)
Mean (Observed)	24	30	29	-66	-40	110	51	64

SD (Observed)	24	31	30	18	17	15	20	48

Correlation	0.5	0.5	0.49	0.01	0.06	0.11	0	0.26

MAE	17.3	21.9	21.6	11.1	11.7	8.06	10.1	43

The mean and standard deviation of the actually observed values are provided as a reference. The correlation is computed between the predicted and observed values for each protein and then averaged.

Since the prediction errors are found to be correlated with the observed ASA states in the data, we explored a relationship between the actually observed values of a feature and the prediction error therein. The results of such an analysis for ASA (Figure S3) are consistent with the previous observation that surface residues are more difficult to predict than buried ones [42-44]. The results for the conformational angles (Figure S4) are also interesting, showing that the prediction error is high for both high and low values of conformational angles. The relationship between the observed values of an ESF and its prediction performance can be explained as follows. A prediction model tries to optimize a performance score and by default assigns equal weights to predictions in each range of ESF values. Thus, if a data is heavily biased towards a given range of observed ESF values, the trained model is also biased likewise, as it tries to model the average behavior of all the samples. Our feature data is biased towards lower values in the case of ASA and towards the middle in the case of conformational angles. Therefore, a trained model performs well in these ranges of observed values and shows higher errors in ranges far from the average of these features.

#### Correlations between prediction errors in individual features

To examine relationships between the predictability of two features, we calculated correlation coefficients between their prediction errors (MAE), shown in Table 3. We observe that significant correlations exist between MAE values for almost all the features, although some of these correlations have a small negative value. First, the three ASA-based features are very strongly correlated with each other, showing that the side chain versus main chain grouping of ASA or the one based on polar and non-polar atoms perform almost identically in the prediction model. However, even if their MAE values are highly correlated, it is worth predicting three separate values, as the side chain and non-polar ASA estimates may provide a more detailed picture of protein structure.

**Table 3:** Coefficients of correlation showing the interdependence between the prediction performances for various structural features.

	MAE (tASA)	MAE (scASA)	MAE (npASA)	MAE (Phi)	MAE (Psi)	MAE (Kappa)	MAE (Alpha)	AUC (HHC)
MAE (tASA)	1	0.93	0.88	0.09	0.1	0.1	0.11	0.19

MAE (scASA)	0.93	1	0.93	0.07	0.08	0.07	0.06	0.21

MAE (npASA)	0.88	0.93	1	0.07	0.08	0.07	0.07	0.2

MAE (Phi)	0.09	0.07	0.07	1	0.46	0.35	0.42	-0.03

MAE (Psi)	0.1	0.08	0.08	0.46	1	0.41	0.47	-0.05

MAE (Kappa)	0.1	0.07	0.07	0.35	0.41	1	0.76	-0.05

MAE (Alpha)	0.11	0.06	0.07	0.42	0.47	0.76	1	-0.04

AUC (HHC)	0.19	0.21	0.2	-0.03	-0.05	-0.05	-0.04	1

The prediction performance for the ASA features is highly correlated with each other. The prediction performance for some conformational angles (e.g., alpha and kappa) is well-correlated, whereas others (e.g., Phi and Kappa) are only weakly correlated.

MAE: Mean absolute error (% ASA or degrees), AUC: Area under the ROC curve; HHC: helix-helix contact.

Second and the most striking observation is that the prediction performance (MAE) of all four conformational angles show a strong positive correlation (R = 0.35 to 0.76) with each other. This observation indicates that the occurrence of residues with far-from-the-average bend angles is also accompanied by far-from-the-average conformational angles alpha, phi and psi, thus all being poorly predicted at the same time.

#### Bend-angle and kink prediction

Helical kinks have been found to be important for various functions of membrane proteins. To estimate if the bend angle prediction in this work can be used for predicting kink angles, we computed kink angles for all the residues (at least 4 residues inside the membrane from both sides) and converted them to a binary value of kinked versus non-kinked at a threshold. We then calculated the AUC of prediction using the bend angle predictions as a score, from which the kinked positions are labeled. Using different thresholds of kink angles, we find the best performance at a 30 degree kink angle, at which the AUC was a modest 57%. There are 141 kinked positions at this cutoff. Thus, at least some critically kinked positions can be identified using this crude approach. For a more accurate prediction of kinked positions, more work is required.

### Relationship between ESF prediction and dynamic structural features (DSFs)

We now explore whether the prediction of the ESFs described above can provide information about the dynamic behavior of TM proteins. We first compared the BNMA (see Methods) with those provided in the PDB files. Table S3 (Additional File 1) shows the correlation between the two sets of B-factors for each protein from the TM76 data set, for which the NMA run was successful and whose B-factors in the PDB file had a non-zero standard deviation. The overall average correlation between the two sets is 0.36, with negative correlations observed in some cases. These results are not surprising, because 1) our data set included medium-resolution structures, where the B-factors may not accurately reflect thermal fluctuations and 2) all our calculations were performed in the isolated chain environments rather than the crystal environments, which usually contained additional protein chains and ligands.

Given that all the analysis in this paper was on the chain level, we decided to use the BNMA as a measure of atomic fluctuations in the TM76 dataset, since the PDB-derived B-factors, though used in similar studies on water-soluble proteins [45], are unsuitable for the reasons described above.

**Figure 2:**
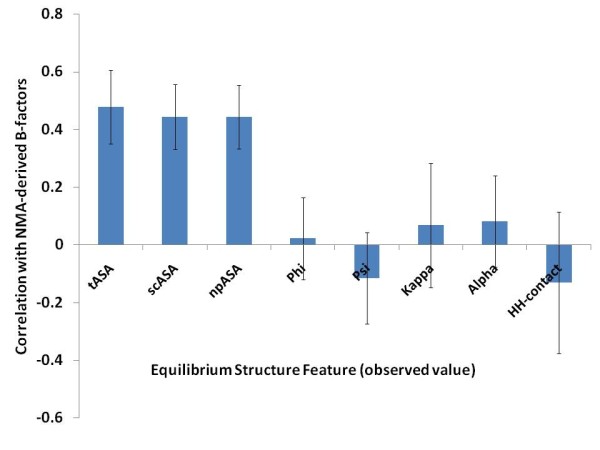
**Correlation between the equilibrium structural feature (ESFs) and the NMA-derived B-factors**. Correlation coefficients were calculated for each protein and averaged for the plot. Error bars represent the standard deviation of the protein-wise correlation coefficients. Predicted B-factors from the El Nemo web server were used for this analysis, which was based on the displacements observed in the 100 lowest-frequency modes.

Figure 2 shows how the individual ESFs are related to the BNMA. We find some correlation of this property with all of the eight features under consideration and hence there is a possibility that BNMA can be predicted from observed or predicted ESFs. We attempted to predict BNMA, first directly from the sequence and then by using observed or predicted ESFs or a combination thereof. Background correlations and correlation between predicted and observed BNMA values are shown in Table 4, along with P-values (see Methods). Only the TM regions were considered for these predictions. Table 4 shows that sequence alone (PSSM information) can only poorly estimate BNMA, whereas predicted ESFs show a slightly better performance. This result shows that a small set of eight features can perform better than a larger set of PSSM-based features, as far as predicting BNMA is concerned. The PSSM-based performance is less accurate than the previously reported performance of the B-factor prediction for water-soluble proteins [45]. The lower performance can be explained by the differences in the environment, in which our BNMA values were calculated (for the isolated single chains) and the PDB-derived B-factors were obtained (in the biological complexes). The isolated chain environment is likely to increase the number of flexible residue positions, making them more difficult to predict.

**Table 4:** Performance of the prediction of NMA-derived B-factors (BNMA) from PSSM, the observed and predicted values and their combination of the equilibrium structural features (ESFs).

Input features	Prediction performance (Correlation with BNMA)	Prediction performance (Correlation with randomized BNMA)	P-value
PSSM (9 residue window) and amino acid composition	0.21	-0.02	1.3e-7

ESFs (Observed)	0.52	0.00	< 2.2e-16

ESFs (Predicted)	0.23	-0.01	4.1e-10

ESFs (Predicted + Observed)	0.46	-0.01	< 2.2e-16

Performance on randomized BNMA is shown for reference and a p-value is calculated using Welsh's t-test on the two sets of protein-wise performance scores (correlation coefficients). The predicted ESFs alone can predict BNMA with reasonable performance.

#### Intra-helical position and BNMA

The residues closer to the membrane boundaries are likely to be more mobile than in the middle. To confirm, we plotted the average BNMA of residues in various slabs along the bilayer normal, using the absolute value of the z-coordinate taken from OPM (Figure S5; Additional File 1). The correlation coefficient between the z-coordinate and BNMA is about 0.23, which is similar to the prediction performance of the predicted ESFs. However, calculating the z-coordinate requires structural information, which is not needed for ESF-based estimates. Further, the correlation between the z-coordinate and BNMA was obtained from a self-fitted model, whereas the values in Table 2 are cross-validation results expected for a new protein. Thus, the use of predicted ESFs for estimating membrane dynamics is likely to result in useful information.

#### Prediction of MD-derived RMSF from various features

We now look at the collective contributions of the ESFs in determining the MD-derived RMSFs. This can be done by developing a neural network for predicting the RMSF from various feature sets. Since there is no central repository for MD trajectories and carrying out MD simulations requires huge computing power, we analyzed the trajectories of only five protein chains in two complexes. Furthermore, our MD simulations were performed for a biological complex, rather than the isolated chains. For these reasons, this part of the analysis should be considered an example application of the method described in this paper, rather than presenting conclusive statistics.

Unlike the previous sections, non-membrane regions were also included for this analysis, because the definition of the membrane-spanning region is not unique in a simulation environment and the number of residues that are expected to be always inside the lipid bilayer was too small to produce meaningful results. Nonetheless, the bias from the inclusion of non-membrane regions could be quantified by calculating the correlation between the RMSF and the residue location in membrane versus non-membrane regions (defined by the native PDB structures used for the simulations). This correlation came out to be 0.48 and it is the minimum correlation for the predictions to be useful.

Table S4 (Additional File 1) shows the performance of various methods of RMSF prediction. As in the case of NMA, the performance of the predicted ESFs is comparable to the baseline value of 0.48 (the correlation without cross-validation). The observed values of ESFs show a much better correlation and a combination of the observed and predicted ESFs improves the results further. This observation gives further evidence for the applicability of predicted and observed values of ESFs to estimate local atomic fluctuations in membrane proteins.

### Web server "HTM One"

To take full advantage of the results presented in this work, we created a web server to predict one-dimensional structural features of proteins. This web server "HTM One" (see "Availability and Requirements") aims to make an integrated prediction of one-dimensional structural features of helical membrane proteins using only an amino acid sequence as the input. It consists of two modules.

#### Prediction module

In this module, the users are required to submit only an amino acid sequence and the server computes a PSSM and pattern data and makes a prediction of the eight one-dimensional structural features of all the residues.

#### Analysis module

In this module, the users upload a PDB formatted file or enter a PDB ID and a chain identifier to be analyzed. The server automatically extracts sequence information and compares the observed structural features of the given protein chain with what would be expected from a purely sequence-based prediction (as in the prediction module). The server returns the observed and predicted values of these features and calculates the prediction errors, which could be used for further analysis, e.g., to estimate atomic displacements of each residue.

In both modules, a local implementation of the neural network based prediction of TM helical residues is carried out to help the users identify which regions to concentrate on (see Methods). Predictions are provided for all the residues, so if the users have their own prior TM segment assignments, they may extract the relevant data from the server output.

## Discussion

This work addressed two key questions about the sequence-based modeling of membrane proteins, i.e., the prediction of one-dimensional structural features and their relationships with conformational flexibility. We were able to demonstrate clearly that a computational model, which predicts multiple structural features in an integrated fashion, works significantly better than the ones trained individually. This is the first effort of its kind and is likely to be helpful in developing even more accurate methods of predicting membrane protein structures.

As a byproduct of this work, we were able to show that kinked positions in membrane proteins are related to the peptide bend angle, even though the latter is not strictly based on the secondary structure definition. Severely kinked positions in helical membrane proteins are significant from a functional perspective and a quick estimate of these positions gives us good insights into the nature of membrane protein dynamics.

The second part of the work aimed at predicting B-factors and here, we clearly showed that within the membrane-spanning regions, predicted and observed one-dimensional structural features led to an effective way of determining flexible helical residue positions. The one-dimensional structural features improved the prediction of flexibility based simply on the residue position with respect to the membrane boundaries. Although the available MD data was insufficient to develop strictly general models, an application of the ESFs-based prediction model showed a good promise for estimating MD-derived dynamics. Hopefully, in future this aspect of the work can be improved. Also, the analysis of local atomic fluctuations undertaken in this work can be extended to large-scale conformational changes, for which work is in progress.

## Conclusion

An integrated system to predict eight one-dimensional structural features of helical membrane proteins was developed. The performance of the integrated system was significantly better than that of the models trained on individual features. The analysis of prediction errors from this system led to a method for estimating NMA-derived fluctuations in proteins. The relationships between the prediction errors and atomic displacements were analyzed by comparing the prediction results with data from NMA and MD simulations. Although sequence information alone has a limited power to estimate atomic displacements, it was shown to improve the structure-based estimates of this property.

## Availability and Requirements

Project name: HTM One

Project home page: http://tardis.nibio.go.jp/netasa/htmone

Operating system: Platform independent

Programming language: C, Perl and bash scripting

License: Free online usage with no restriction or warranty

## Authors' contributions

SA designed and implemented this study in consultation with KM. YHS provided data sets and benchmark results. YS and TM provided MD data. SA and KM prepared manuscript and all authors read and approved of it.

## Supplementary Material

Additional file 1 **additional-data.pdf (PDF format, requires acrobat reader)**. Click here for file
