# Supplementary material for: Integrated prediction of one-dimensional structural features and their relationships with conformational flexibility in helical membrane proteins
Source: BMC Bioinformatics. 2010 Oct 27;11:533. doi: 10.1186/1471-2105-11-533 (PMC3247134; doi:10.1186/1471-2105-11-533)
Supplement: Additional file 1 — additional-data.pdf (PDF format, requires acrobat reader). [file 1471-2105-11-533-S1.PDF]

Figure S1. Distribution of various structural features in selected proteins (membrane spanning regions).

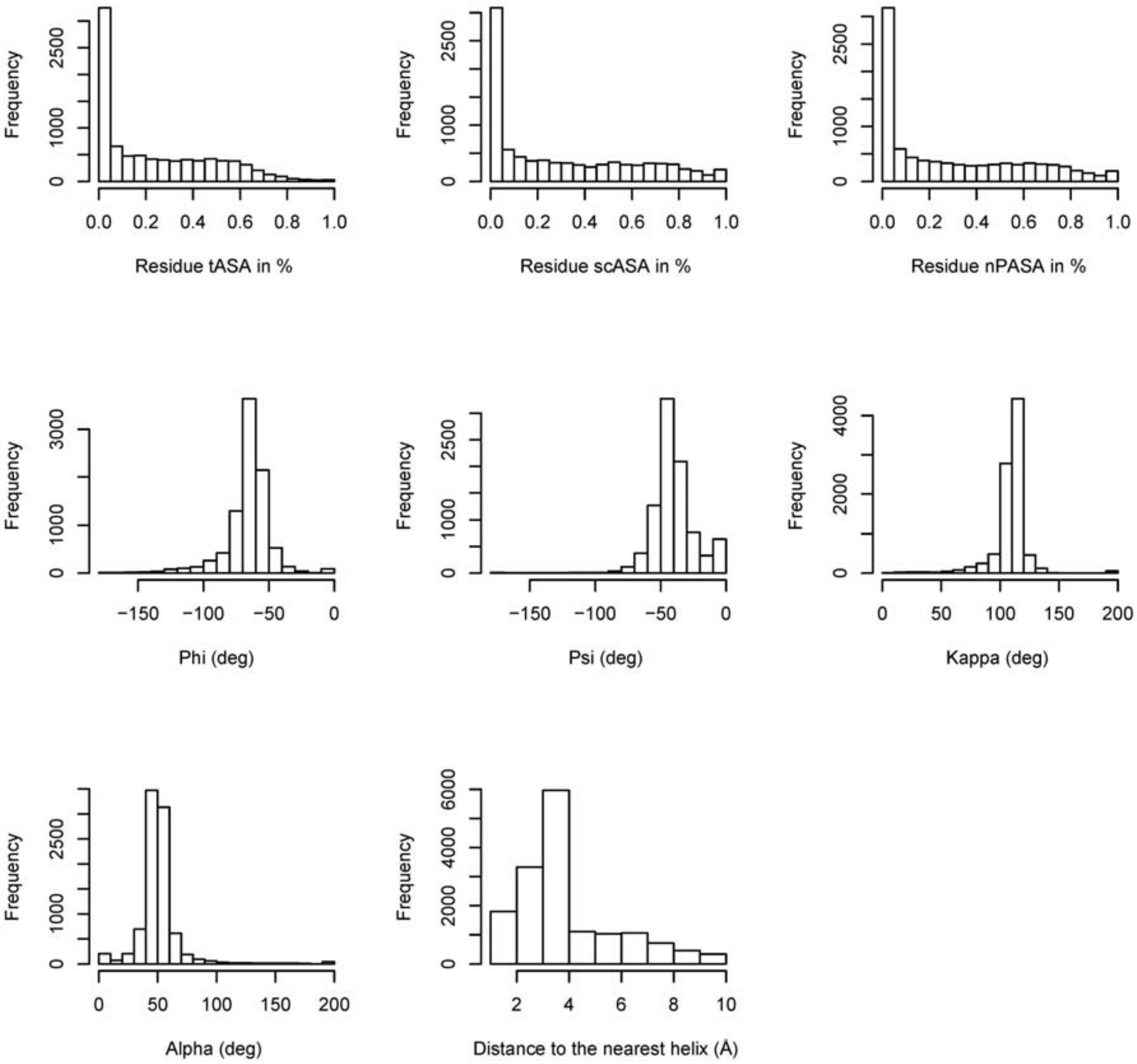

Figure S2. Mean absolute error of predicted solvent accessibility from ASAP and MPRAP web servers in comparison to the proposed method (HTM One). Although, MPRAP performs slightly better than LOO cross-validated HTM One, it may be because some proteins used for benchmarking are already present in the trained model, used in the web server. This likelihood is supported by the best performance of HTM Web server (based on a self-consistent prediction model). Relatively lower performance of ASAP may also be caused by the same reason, as the method was developed much earlier and is likely based on fewer proteins.

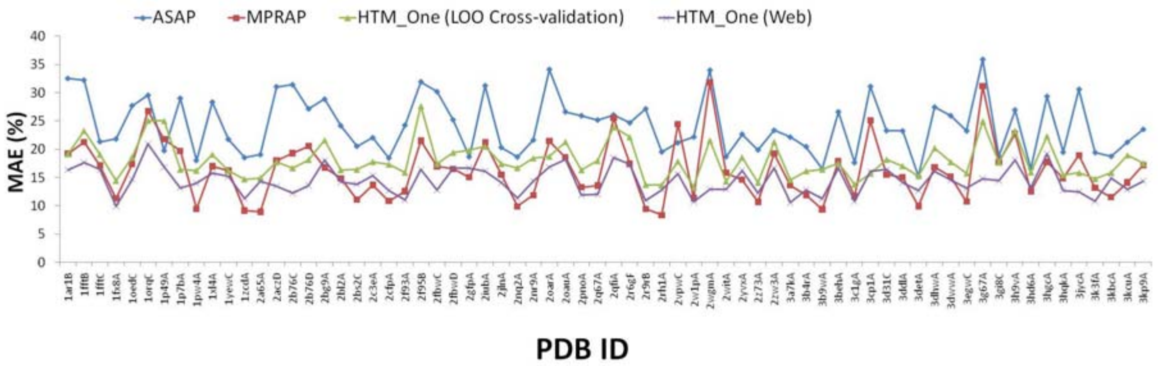

Figure S3. Prediction performance of HTMOne for helix-helix contact prediction. A similar web server RHYTHM gives only a binary prediction score from which sensitivity and specificity were computed, shown by the interaction of axis lines. Since, RHYTHM requires membrane protein annotation as channel protein or coiled coil as an input to the web server, and HTMOne requires none, all predictions from RHYTHM were made by assuming proteins to be coild-coil for a fair comparison, which may explain lower than expected performance.

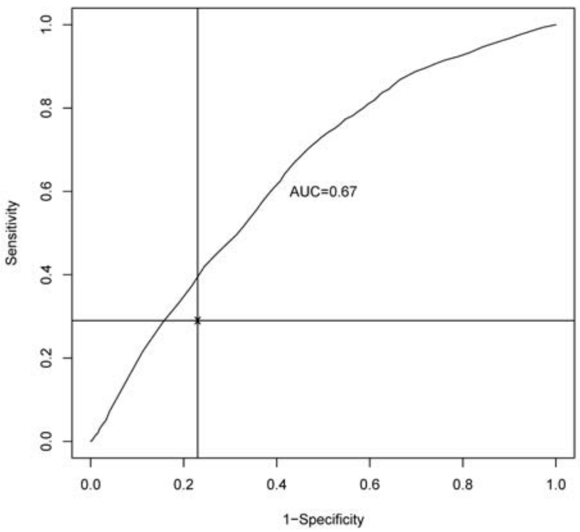

Figure S4. Prediction absolute error in residue-wise (total) solvent accessibility as a function of observed values. As expected, performance is lower for highly exposed residues and best near the mean values (tASA ~24%).

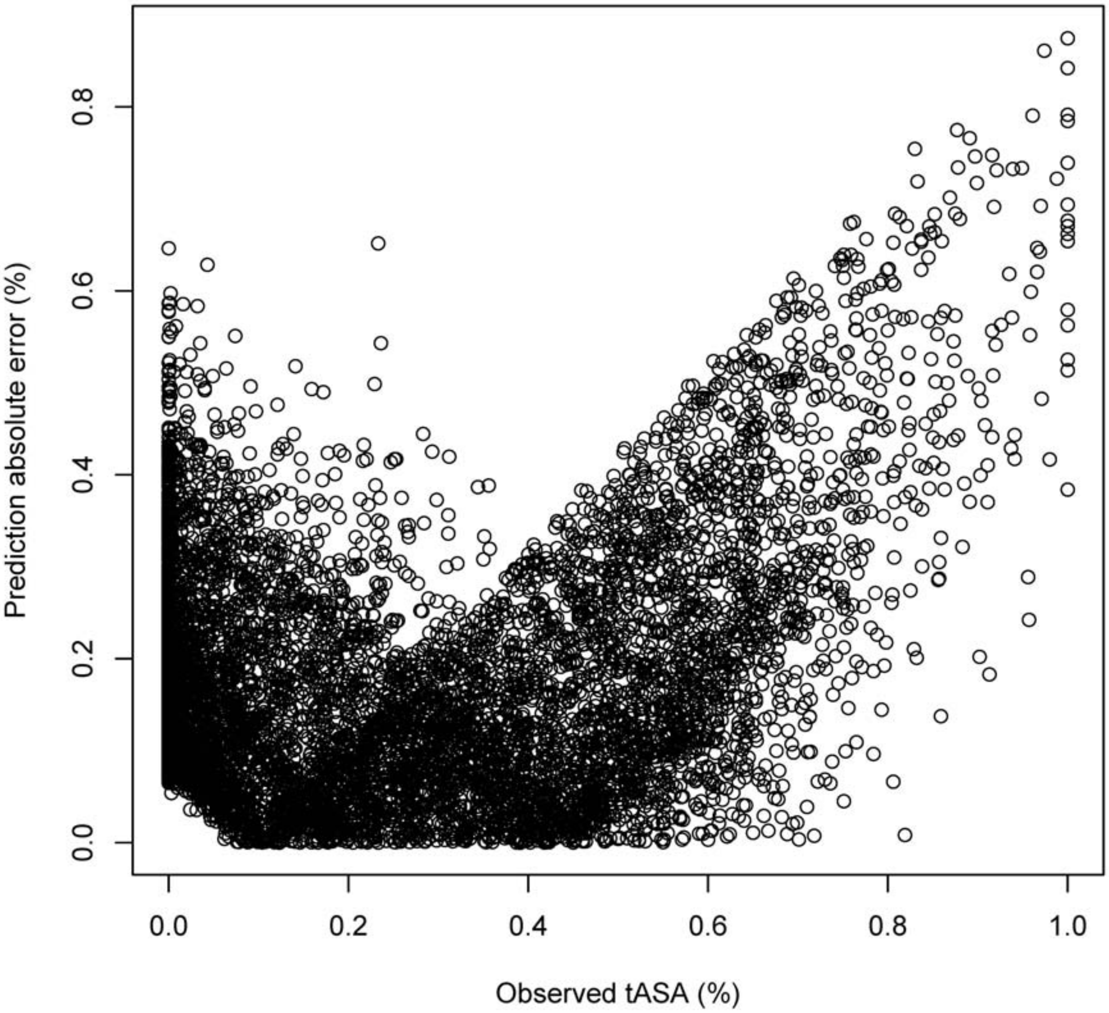

Figure S5. Residue-wise prediction absolute error as a function of actual value of the dihedral, bend and torsion angles. As in the case of ASA, best performance comes close to the mean values of these angles and falls on both sides in a more symmetric way than ASA (Figure 5).

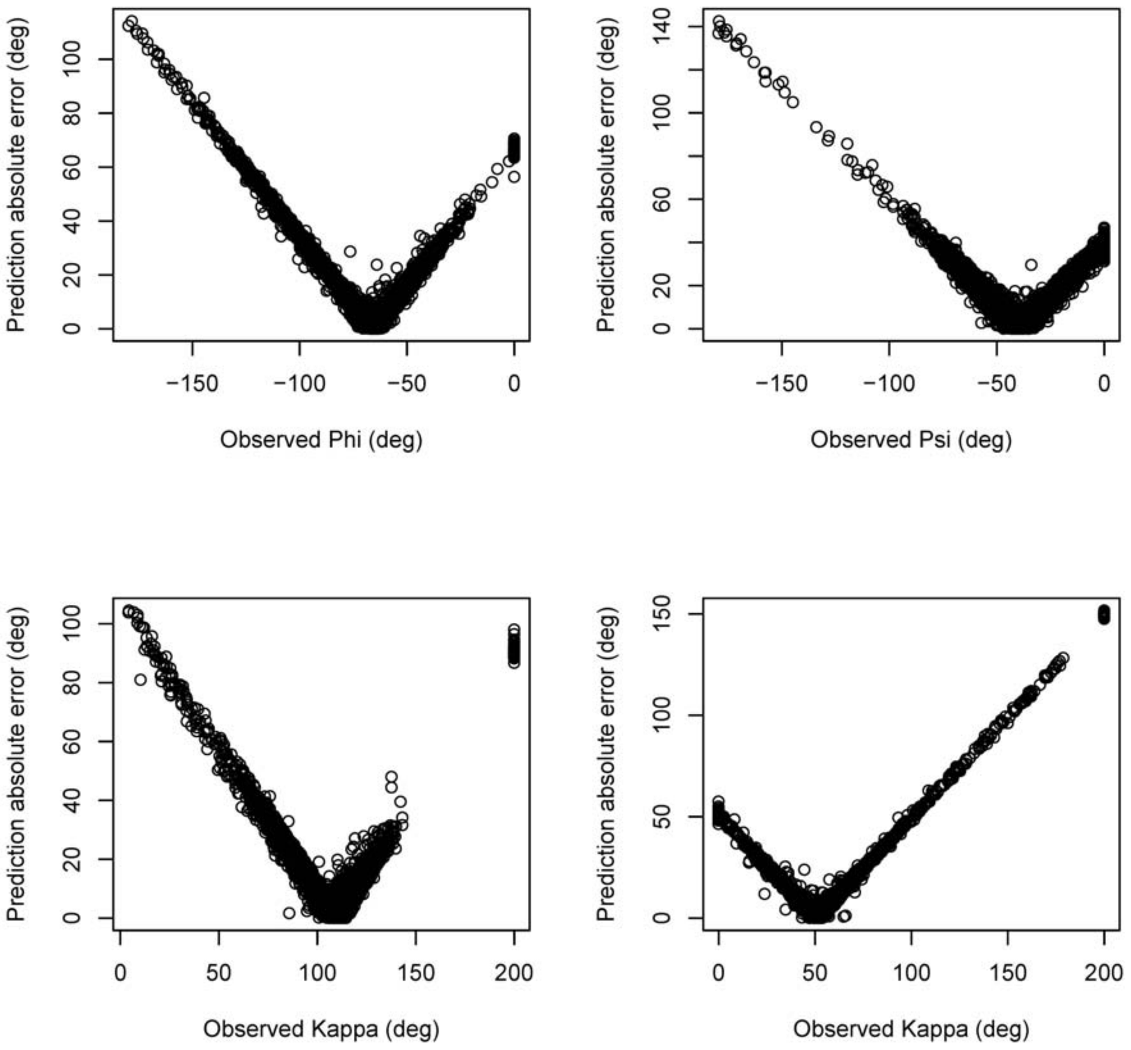

Table S1. Correlation between residue-wise values of eight structural features (ESFs) considered in this work.

Feature	tASA	scASA	npASA	Phi	Psi	Kappa	Alpha	HHC
tASA	1	0.98	0.96	0.02	-0.05	0.05	-0.01	-0.34
scASA	0.98	1	0.98	0	-0.02	0.04	-0.01	-0.36
npASA	0.96	0.98	1	0	-0.02	0.05	-0.01	-0.36
Phi	0.02	0	0	1	-0.25	-0.08	0	0.01
Psi	-0.05	-0.02	-0.02	-0.25	1	0.26	-0.26	-0.03
Kappa	0.05	0.04	0.05	-0.08	0.26	1	-0.06	-0.09
Alpha	-0.01	-0.01	-0.01	0	-0.26	-0.06	1	0.02
HHC	-0.34	-0.36	-0.36	0.01	-0.03	-0.09	0.02	1

Table S2. Protein-wise ESFs prediction performance of integrated prediction model.

PDBID	TM regions	MAE (tASA) (%)	MAE (scASA) (%)	MAE (npASA) (%)	MAE (Phi) (%)	MAE (Psi) (%)	MAE (Kappa) (deg)	MAE (Alpha) (deg)	AUC (HHC) (deg)
3g5uA	1(45-68); 2(114-132); 3(184-203); 4(215-234); 5(291-310); 6(332-349); 7(708-728); 8(753-773); 9(829-847); 10(852-872); 11(933-953); 12(973-990)	17.47	22.92	22.35	17.65	19.98	6.39	8.4	60.19
2gifA	1(11-27); 2(341-357); 3(366-386); 4(395-410); 5(443-458); 6(469-489); 7(542-558); 8(874-890); 9(899-919); 10(927-943); 11(976-992); 12(1001-1019)	19.09	23.29	22.43	8.56	9.28	6.46	7.89	72.97
3kg2A	1(522-541); 2(577-586); 3(597-618); 4(792-811)	23.78	31.93	33.5	8.6	12.24	7.48	10.93	70.01
1p49A	1(181-205); 2(212-233)	25.02	33.63	34.99	7.84	8.12	8.09	6.37	64.52
3dtuA	1(25-53); 2(99-125); 3(134-158); 4(190-214); 5(227-251); 6(278-302); 7(311-334); 8(348-371); 9(378-403); 10(415-440); 11(449-473); 12(494-520)	14.61	18.48	18.31	8.63	9	7.11	8.3	58.09
2witA	1(61-76); 2(99-116); 3(143-158); 4(183-201); 5(237-258); 6(283-299); 7(301-322); 8(368-385); 9(399-416); 10(456-477); 11(490-506); 12(519-538)	14.3	17.78	17.52	11.33	13.3	19.9	26.73	60.55
2a65A	1(16-35); 2(41-63); 3(89-124); 4(167-185); 5(191-215); 6(238-265); 7(280-299); 8(340-367); 9(377-394); 10(400-425); 11(448-472); 12(485-503)	14.93	18.23	17.37	8.1	8.9	7.2	8.94	69.36

2r6gF	1(17-32); 2(44-60); 3(66-84); 4(282-306); 5(314-329); 6(370-391); 7(426-440); 8(493-504)	22.16	27.45	26.8	9.94	11.84	8.74	12.64	69.09
2jlnA	1(31-52); 2(60-76); 3(110-134); 4(140-157); 5(164-181); 6(208-225); 7(254-271); 8(301-320); 9(342-357); 10(359-372)	17.44	21.78	21.35	12.03	11.94	8.56	10.29	56.54
3detA	1(36-61); 2(81-101); 3(125-142); 4(144-159); 5(178-191); 6(193-204); 7(215-233); 8(250-281); 9(289-306); 10(335-351); 11(353-371); 12(390-403); 13(405-419); 14(421-442)	15.28	19.44	19.17	13.73	12.23	9.57	12.77	73.32
2rh1A	1(32-59); 2(70-95); 3(105-129); 4(151-175); 5(194-220); 6(275-299); 7(303-327)	13.66	17.24	17.18	8.12	9	7.97	9.19	66.29
2yvxA	1(282-305); 2(318-336); 3(356-381); 4(388-409); 5(423-443)	18.56	23.72	22.41	14.99	16.86	7.77	10.02	63.4
3gi8C	1(15-33); 2(42-59); 3(89-113); 4(124-140); 5(151-170); 6(191-206); 7(225-242); 8(313-326); 9(334-352); 10(357-375); 11(380-397)	17.67	22.6	22.41	10.79	11.26	9.53	10.99	64.58
1pw4A	1(30-52); 2(64-84); 3(91-111); 4(122-141); 5(160-179); 6(190-208); 7(257-280); 8(291-310); 9(323-342); 10(347-369); 11(387-408); 12(414-433)	16.22	20.56	20.37	11.62	10.62	8.98	11.42	67.55
3hqkA	1(14-27); 2(47-60); 3(89-105); 4(129-141); 5(147-162); 6(201-211); 7(228-241); 8(285-298); 9(328-339); 10(354-	15.45	19.8	20.07	11.37	11.34	8.47	10.24	61.99

	369); 11(390-406); 12(410-423)								
2cfpA	1(9-31); 2(47-67); 3(74-91); 4(109-129); 5(144-160); 6(170-188); 7(223-244); 8(260-279); 9(290-309); 10(314-333); 11(350-370); 12(382-400)	17.34	22.31	22.04	20.44	19.23	9.88	12.85	65.25
3kbcA	1(14-29); 2(43-66); 3(94-110); 4(131-139); 5(150-161); 6(201-217); 7(225-242); 8(313-326); 9(334-352); 10(357-375); 11(380-397)	15.87	20.57	19.98	13.82	13.99	9.79	12.35	61.95
3hgcA	3hgcA:1(51-75); 2(423-448)	22.31	30.39	28.15	20.77	18.83	16.88	14.14	51.8
3hd6A	3hd6A:1(12-28); 2(61-78); 3(89-109); 4(125-142); 5(149-169); 6(178-196); 7(221-238); 8(250-267); 9(282-297); 10(304-321); 11(339-358); 12(395-416)	15.92	20.56	20.12	7.66	7.5	6.98	8.87	70.97
2r9rB	2r9rB:1(164-182); 2(223-242); 3(252-272):4(289-307); 5(322-345); 6(382-402)	13.73	16.84	16.93	8.47	8.15	5.07	6.65	81.84
1zcdA	1zcdA:1(12-27); 2(63-80); 3(98-115); 4(125-141); 5(157-175); 6(184-199); 7(205-220); 8(223-237); 9(255-273); 10(295-313); 11(330-348); 12(363-380)	14.72	18.83	18.37	17.84	17.38	9.79	13.56	70.41
2gfpA	2gfpA:1(12-32); 2(43-59); 3(72-88); 4(99-115); 5(137-153); 6(158-177); 7(215-231); 8(238-255); 9(272-288); 10(290-304); 11(332-349); 12(355-370)	19.81	25.59	24.83	10.32	16.13	8.58	12.65	54.53
2bg9A	2bg9A:1(219-236); 2(243-260); 3(282-	21.62	27.95	27.76	10.26	12.16	6.18	10.38	60.54

	300); 4(409-426)								
3c1gA	3c1gA:1(15-31); 2(46-64); 3(102-118); 4(127-147); 5(161-178); 6(202-219); 7(231-248); 8(261-276); 9(282-299); 10(314-332); 11(353-377)	13.76	17.81	17.35	9.78	9.58	6.05	8.51	77.64
3b9wA	3b9wA:1(19-36); 2(45-63); 3(82-99); 4(105-122); 5(141-159); 6(179-195); 7(209-228); 8(237-254); 9(260-277); 10(293-313); 11(323-347)	16.42	20.82	20.61	7.34	7.94	7.18	9.12	76.01
2z73A	2z73A:1(36-57); 2(72-94); 3(110-131); 4(153-172); 5(200-221); 6(263-282); 7(298-317)	14.11	17.49	17.2	9.49	10.01	5.94	7.52	71.56
3k3fA	3k3fA:1(33-46); 2(48-60); 3(80-92); 4(98-114); 5(130-144); 6(195-205); 7(212-229); 8(239-251); 9(266-285); 10(291-304)	14.78	19	18.52	8.86	8.61	7.55	9.36	69.84
2iubA	2iubA:1(294-310); 2(331-346)	20.5	25.93	25.49	15.33	16.13	5.74	9.34	65
3h9vA	3h9vA:1(35-55); 2(332-360)	23.28	29.77	31.8	11.77	13.17	13.69	17.01	60.76
3jycA	3jycA:1(78-104); 2(156-181)	15.83	19.72	20.37	13.04	13.27	4.28	7.79	69.86
3eamA	3eamA:1(199-218); 2(222-240); 3(256-275); 4(292-313)	19.18	24.25	23.84	10.34	9.48	7.1	6.92	67.23
2nq2A	2nq2A:1(11-24); 2(66-83); 3(93-107); 4(122-138); 5(152-167); 6(197-212); 7(242-257); 8(259-268); 9(284-301); 10(311-326)	16.7	21.94	22.05	9.23	9.86	6.51	8.86	62.63
1lnqA	1lnqA:1(22-38); 2(72-95)	14.17	20.86	20.46	21.81	21.93	8.21	9.95	79.4
2c3eA	2c3eA:1(5-30); 2(73-100); 3(109-138); 4(173-203); 5(207-	17.79	22.22	21.89	13	11.52	10.46	13.59	61.64

	235); 6(273-293)								
1xl4A	1xl4A:1(45-71); 2(108-135)	19.08	25.61	25.18	6.5	8.63	4.77	5.95	84.34
2qfiA	2qfiA:1(18-36); 2(42-57); 3(83-102); 4(122-139); 5(147-165); 6(178-199)	23.91	28.94	28.18	35.52	30.53	20.67	28.17	61.86
2wcdA	2wcdA:1(21-45); 2(166-185); 3(192-213)	25.03	33.42	30.35	12.68	15.23	11.28	10.98	53.42
2w1pA	2w1pA:1(48-66); 2(87-104); 3(131-151); 4(170-189); 5(200-216); 6(243-260)	13.32	16.08	15.94	5.63	6.25	5.24	6.15	71.74
3a7kA	3a7kA:1(37-54); 2(70-87); 3(121-15); 4(152-169); 5(175-192); 6(217-232); 7(244-262)	14.61	18.23	18.28	5.19	8.36	5.47	8	74.28
3kp9A	3kp9A:1(22-40); 2(65-89); 3(104-124); 4(130-148); 5(161-181)	17.52	22.12	21.24	11.97	12.25	8.75	10.65	67.62
1p7bA	1p7bA:1(61-85); 2(122-145)	16.41	21.19	22.43	19	21.34	4.87	6.29	87.33
1fftB	1fftB:1(50-65); 2(90-104)	23.29	30.91	30.72	24	18.34	8.12	10.17	67.08
1fx8A	1fx8A:1(11-28); 2(43-60); 3(86-104); 4(149-167); 5(179-195); 6(234-255)	14.44	17.37	17.6	6.86	7.58	5.6	6.66	78.97
2bs2C	2bs2C:1(29-50); 2(77-99); 3(125-151); 4(167-192); 5(206-232)	16.39	20.94	20.75	6.08	7.61	6.6	7.85	72.82
2oauA	2oauA:1(32-58); 2(70-89); 3(91-104)	21.32	28.33	28.67	4.96	7.18	7.5	7.38	57.7
1ar1B	1ar1B:1(36-55); 2(80-97)	19.17	24.51	24.35	8.86	9.49	3.17	5.18	67.92
3ddlA	3ddlA:1(20-41); 2(46-65); 3(92-112); 4(121-141); 5(149-173); 6(188-209); 7(231-252)	17.04	20.82	20.28	7.98	8.58	5.62	7.38	69.58
3kcuA	3kcuA:1(36-59); 2(63-83); 3(115-141); 4(146-179); 5(193-204); 6(210-226);	18.94	24.19	24.49	9.67	10.17	8.37	11.14	56.41

	7(245-269)								
2vpwC	2vpwC:1(17-37); 2(50-67); 3(91-106); 4(115-132); 5(148-164); 6(176-190); 7(208-222); 8(227-242)	17.83	23.07	22.3	8.96	10.68	5.35	5.9	69.97
3d31C	3d31C:1(18-39); 2(62-83); 3(96-116); 4(141-159); 5(198-215); 6(246-260)	18.18	22.07	22.28	14.85	14.03	9.14	11.64	68.95
3egwC	3egwC:1(5-28); 2(48-73); 3(87-114); 4(125-145); 5(183-204)	15.77	20	18.89	7.48	9.96	8.38	8.61	74.3
1orqC	1orqC:1(21-52); 2(57-80); 3(151-171); 4(209-236)	25.08	31.91	29.96	15.3	15.71	6.99	8.72	62.04
3behA	3behA:1(16-29); 2(38-53); 3(81-93); 4(96-108); 5(134-150); 6(187-203)	17.49	22.32	22.32	6.34	9.52	9.96	10.62	69.73
2f93A	2f93A:1(7-27); 2(35-55); 3(71-90); 4(94-115); 5(125-146); 6(161-180); 7(193-214)	15.89	19.52	18.38	6.16	6.82	5.34	5.67	75.17
3b4rA	3b4rA:1(18-32); 2(41-57); 3(97-113); 4(126-147); 5(170-186); 6(191-205)	16.15	20.08	20.54	12.32	13.66	6.22	8.58	66.47
3g67A	3g67A:1(125-146); 2(151-170)	24.96	31.41	30.89	6.82	8.93	5.88	4.98	59.88
3dhwA	3dhwA:1(13-34); 2(56-75); 3(90-105); 4(151-164); 5(191-207)	20.22	25.02	23.85	6.9	9.19	9.65	12.81	57.38
2zw3A	2zw3A:1(25-50); 2(66-89); 3(140-159); 4(183-208)	21.29	25.78	24.86	17.32	16.68	8.1	13.75	65.86
2nr9A	2nr9A:1(11-24); 2(64-78); 3(88-104); 4(115-125); 5(144-157); 6(167-179)	18.41	23.46	22.68	8.57	9.05	5.76	7.27	58.54
1yewC	1yewC:1(52-67); 2(93-108); 3(139-155); 4(175-190)	16.2	20.25	20.78	11.48	12.6	6.66	7.61	71.94
1fftC	1fftC:1(31-44); 2(68-80); 3(103-116);	19.08	24.84	25.18	19.35	16.35	6.31	9.81	68.64

	4(143-159); 5(182-196)								
2bl2A	2bl2A:1(16-35); 2(61-78); 3(93-110); 4(137-154)	16.33	21.44	21.3	5.82	7.12	6.59	5.75	82.66
2pnoA	2pnoA:1(8-21); 2(58-71); 3(79-91); 4(111-124)	16.31	20.34	20.43	7.63	7.06	3.1	3.86	63.07
3dwwA	3dwwA:1(16-40); 2(68-90); 3(98-119); 4(126-146)	17.77	21.5	22.35	11.33	15.3	8.14	8.67	64.65
2fbwC	2fbwC:1(35-62); 2(87-113); 3(119-138)	17.41	21.73	19.89	7.68	8.23	5.72	7.97	70.64
2b76C	2b76C:1(27-47); 2(66-91); 3(107-126)	16.68	22.21	22.58	13.32	10.06	5.5	6.17	65.73
2aczC	2aczC:1(26-49); 2(68-93); 3(109-129)	18.2	22.77	21.83	8.63	10.3	6.06	9.23	76.55
1oedC	1oedC:1(227-250); 2(258-279); 3(291-313); 4(456-480)	18.63	23.63	24.85	6.96	7.41	5.9	5.9	66.76
2oarA	2oarA:1(19-47); 2(67-89)	18.71	24.17	24.82	6.68	6.66	11.32	12.92	62.28
2b76D	2b76D:1(13-38); 2(62-86); 3(94-116)	18.14	22.89	22.48	16.72	18	6.76	10.42	65.51
2aczD	2aczD:1(16-39); 2(55080); 3(89-113)	17.76	21.48	20.66	8.18	10.18	2.92	3.8	77.53
2q67A	2q67A:1(26-42); 2(76-94)	17.95	21.65	20.74	4.52	4.72	2.67	2.76	80.84
2fbwD	2fbwD:1(9-28); 2(35-56); 3(66-85)	19.41	26.88	27.25	6.14	6.57	4.3	4.62	66.12
2wgmA	2wgmA:1(11-32); 2(62-85)	21.58	28.95	28.7	8.95	10.47	12.3	10.87	72.12
3cp1A	3cp1A:1(15-39); 2(56-79)	15.72	17.08	22.12	6.14	7.26	7.21	2.72	62.67
2f95B	2f95B:1(27-38); 2(65-79)	27.66	34.24	35.37	6.15	5.84	16.58	19.4	36.84

Table S3. Protein-wise correlation between single-chain NMA-derived B-factors and those read from the PDB files of full complex structure.

PDBID	Corr (BNMA and Crystal B-factor)	PDBID	Corr (BNMA and Crystal B-factor)	PDBID	Corr (BNMA and Crystal B-factor)	PDBID	Corr (BNMA and Crystal B-factor)	PDBID	Corr (BNMA and Crystal B-factor)	PDBID	Corr (BNMA and Crystal B-factor)
2a65A	0.62	3kbcA	0.17	3k3fA	0.2	2wcdA	0.42	3ddlA	0.72	3dhwA	0.26
2r6gF	0.54	3hgcA	0.65	2iubA	0.6	2w1pA	0.66	3kcuA	0.77	2zw3A	0.36
2jlnA	0.43	3hd6A	0.37	3h9vA	0.7	3a7kA	0.71	2vpwC	-0.19	2nr9A	0.2
3detA	0.47	2r9rB	0.04	3jycA	0.57	3kp9A	0.07	3d31C	0.22	1yewC	-0.1
2rh1A	0.33	1zcdA	0.24	3eamA	0.58	1p7bA	0.6	3egwC	-0.11	1fftC	NA
2yvxA	0.55	2gfpA	NA	2nq2A	0.68	1fftB	NA	1orqC	0.34	2bl2A	0.71
3gi8C	0.06	2bg9A	NA	1lnqA	0.3	1fx8A	0.61	3behA	0.08	2pnoA	0.75
1pw4A	0.13	3c1gA	0.14	2c3eA	0.49	2bs2C	-0.11	2f93A	NA	3dwwA	
3hqkA	-0.03	3b9wA	0.51	1xl4A	0.64	2oauA	0.66	3b4rA	0.22	2fbwC	-0.04
2cfpA	0.8	2z73A	0.59	2qfiA	0.27	1ar1B	0.47	3g67A	0.79	2b76C	-0.17
1oedC	NA	2b76D	0.22	2aczD	0.69	2q67A	0.33	2fbwD	0	2aczC	0.75
2oarA	0.47	3cp1A	0.67	2f95B	0.61	2wgmA	0.53				
**Average:**	**0.38**										

Table S4. Performance of predicting MD-derived RMSF in 5 protein chains MD-trajectory of two complexes. Results include non-membrane regions due to insufficient data. TM annotation alone is capable of a predicting RMSF with a correlation ~0.48, which is better than predicted ESFs alone. However, all other performances are better than this score and combining observed and predicted ESFs can notably improve performance of RMSF-prediction models. Statistical significance is low due to insufficient data, and hence the results are somewhat tentative, but also intuitive.

Inputs for MD RMSF Prediction	Correlation with randomized values	Correlation with observed RMSF values
Observed ESFs	0.07	0.63
Predicted ESFs	0.02	0.44
Observed ESFs+ Predicted ESFs	-0.01	0.67
